# Supplementary material for: Identification of photoperiod-induced specific miRNAs in the adrenal glands of Sunite sheep (Ovis aries)
Source: Front Vet Sci. 2022 Jul 22;9:888207. doi: 10.3389/fvets.2022.888207 (PMC9354845; doi:10.3389/fvets.2022.888207)
Supplement: Supplementary file 1 [file Data_Sheet_1.ZIP › Supplementary file/miRDeep2 file.zip!/miRDeep2 file/Know_Structure/SPLP42A1/oar-mir-22.pdf]

miRBase precursor : oar-mir-22
Total read count : 93131
oar-miR-22-3p read count : 87476
remaining reads : 5655

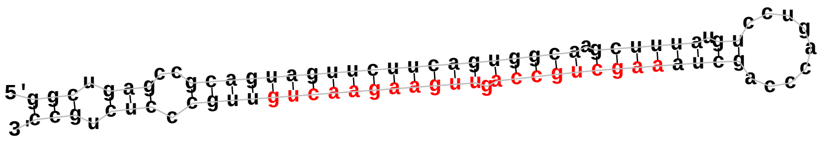

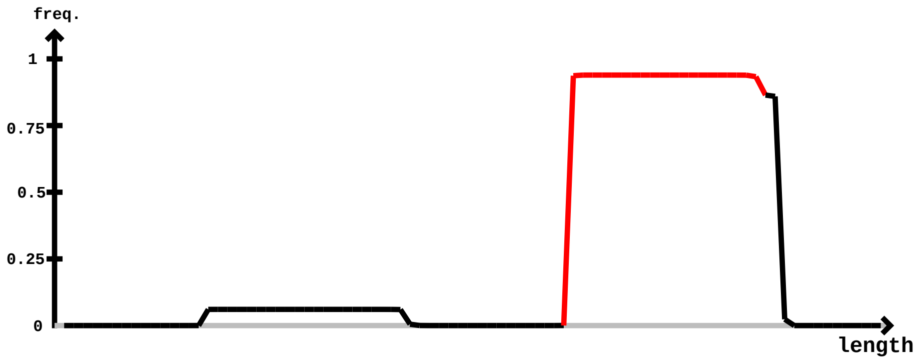

oar-miR-22-3p

```
5'- ggcugagccgcaguaguucuucaguggcaagcuuuauguccugacccagcuaaagcugccaguugaagaacuguugcccucugcc   -3'    exp
    (((.(((..((((((((((((((((((((.((((((.((.........))))))))))))).))))))))))))))).))).)))    reads      mm         sample
    .............uaguucuucaguggcaagcuuu..................................................    7          0          seq
    ..............aguucuucaguggcaagA.....................................................    1          1          seq
    ..............aguucuucaguggcaagU.....................................................    1          1          seq
    ..............aguucuucaguggcaagc.....................................................    1          0          seq
    ..............aguucuucaguggcaagcu....................................................    5          0          seq
    ..............aguucuucaguggcaagcuu...................................................    54         0          seq
    ..............aguucuucaguggcaagcuuu..................................................    5088       0          seq
    ..............aguucuucaguggcaagGuuu..................................................    1          1          seq
    ..............aguucuucaguggcaagcuuua.................................................    456        0          seq
    ..............aguucuucaguggcaagcuuuau................................................    31         0          seq
    ...............guucuucaguggcaagcuuu..................................................    10         0          seq
    ..................................................uaaagcugccaguugaagaacu.............    1          0          seq
    ...................................................aaagcugccaguugaagaac..............    1          0          seq
    ...................................................aaagcugccaguugaagaacu.............    6          0          seq
    ...................................................aaagcugccaguugaagaacugu...........    18         0          seq
    ...................................................aaagcugccaguugaagaacuguu..........    1          0          seq
    ....................................................aagcugccaguugaaga................    11         0          seq
    ....................................................aagcugccaguugaagaa...............    24         0          seq
    ....................................................aagcugccaguugaagaac..............    534        0          seq
    ....................................................aagcugccaguugaagaacu.............    6392       0          seq
    ....................................................aagcugccaguugaagaacug............    456        0          seq
    ....................................................aagcugccaguugaagaacugu...........    77623      0          seq
    ....................................................aagcugccaguugaagaacuguu..........    2181       0          seq
    ....................................................aagcugccaguugaagaacuguug.........    3          0          seq
    .....................................................agcugccaguugaagaacu.............    10         0          seq
    .....................................................agcugccaguugaagaacugu...........    197        0          seq
    .....................................................agcugccaguugaagaacuguu..........    14         0          seq
    ......................................................gcugccaguugaagaacugu...........    3          0          seq
    ......................................................gcugccaguugaagaacuguu..........    1          0          seq
```
